# Theoretical Distribution of the Ammonia Binding Energy at Interstellar Icy Grains: A New Computational Framework

**DOI:** 10.1021/acsearthspacechem.2c00040

**Published:** 2022-06-02

**Authors:** Lorenzo Tinacci, Auréle Germain, Stefano Pantaleone, Stefano Ferrero, Cecilia Ceccarelli, Piero Ugliengo

**Affiliations:** † Dipartimento di Chimica, Università degli Studi di Torino, via P. Giuria 7, 10125 Torino, Italy; ‡ Institut de Planétologie et d’Astrophysique de Grenoble (IPAG), 38000 Grenoble, France; § Dipartimento di Chimica, Biologia e Biotecnologie, Università degli Studi di Perugia, 06123 Perugia, Italy; ∥ Departament de Quimica, Universitat Autònoma de Barcelona, 08193 Bellaterra, Catalonia Spain

**Keywords:** amorphous water ice, xTB-GFN2, ONIOM, DLPNO, B97D3, NH_3_ adsorption, NH_3_ binding energy

## Abstract

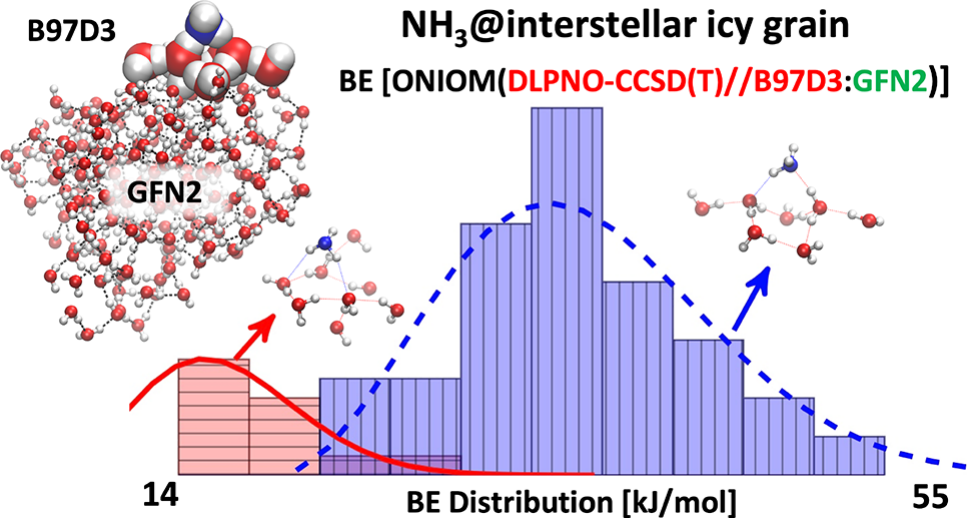

The binding energies (BE) of molecules on the interstellar grains are crucial in the chemical evolution of the interstellar medium (ISM). Both temperature-programmed desorption (TPD) laboratory experiments and quantum chemistry computations have often provided, so far, only single values of the BE for each molecule. This is a severe limitation, as the ices enveloping the grain mantles are structurally amorphous, giving rise to a manifold of possible adsorption sites, each with different BEs. However, the amorphous ice nature prevents the knowledge of structural details, hindering the development of a common accepted atomistic icy model. In this work, we propose a computational framework that closely mimics the formation of the interstellar grain mantle through a water by water accretion. On that grain, an unbiased random (but well reproducible) positioning of the studied molecule is then carried out. Here we present the test case of NH_3_, a ubiquitous species in the molecular ISM. We provide the BE distribution computed by a hierarchy approach, using the semiempirical xTB-GFN2 as a low-level method to describe the whole icy cluster in combination with the B97D3 DFT functional as a high-level method on the local zone of the NH_3_ interaction. The final ZPE-corrected BE is computed at the ONIOM(DLPNO-CCSD(T)//B97D3:xTB-GFN2) level, ensuring the best cost/accuracy ratio. The main peak of the predicted NH_3_ BE distribution is in agreement with experimental TPD and computed data in the literature. A second broad peak at very low BE values is also present, which has never been detected before. It may provide the solution to a longstanding puzzle about the presence of gaseous NH_3_ also observed in cold ISM objects.

## Introduction

Interstellar dust grains in cold (∼10 K) molecular clouds are made up of sub-micrometer-sized refractory cores (mainly silicates and carbonaceous material), on top of which water molecules are formed *in situ* through reactions involving hydrogen and oxygen.^1−5^ Eventually, this process leads to the accretion of a thick (made up of more than 100 layers: e.g. Taquet et al.^6^) amorphous icy mantle. At the same time, other atoms and molecules formed in the gas phase can condense and be adsorbed onto the grain mantle, where they may diffuse and react on the icy surface, enriching the chemical composition of the grain mantle.

The vast majority of the species frozen or trapped on the grain mantles are only observable when they are released into the gas phase either in warm (⩾100 K) regions, such as hot cores/corinos via thermal desorption,^7−9^ or in shocked regions, via sputtering of the mantles.^10−12^ All of the processes mentioned above, adsorption and diffusion as well as desorption, are governed by a key parameter, the so-called binding energy (BE): namely, the strength of a species to remain glued to the surface. Since BE has an exponential dependence on the expressions of astrochemical models that describe the above processes, its estimation with a good level of accuracy is crucial for our knowledge of chemical evolution of whatever species.^13^ This fundamental piece of information can be obtained via either theoretical or experimental approaches.

Usually, estimates of PES via theoretical methods involve computations using a molecular mechanics force-field approach and/or rigorous quantum mechanical methods. In both cases, an atomistic model of the icy grain is needed and the BE is computed via a supermolecular approach: namely, computing the difference between the energy of the adsorbate interacting with the icy grain and the energies of the free adsorbate plus the original icy grain. Despite this simple definition, the final BE value can be affected by many factors, from both modeling and methodological points of view. To start with, the computerized icy model is usually ill-defined, as the structure of the interstellar ice is poorly known. Therefore, a variety of models to simulate the ice-species adsorption has been proposed in the literature, from just a single water molecule up to periodic models of either crystalline or amorphous water ice.^14−16^ Due to the difficulty of simulating the icy grain accretion by *in situ* water formation, all of the models so far have been constructed by assembling a variety of already formed water molecules interacting through hydrogen bonds.^17,18^ This may have serious consequences on the final ice structure, as the fraction of water formation energy transferred to the grain can affect its final structural features much more than the mere hydrogen-bond interaction between the water molecules.^19^ In addition, it has been shown theoretically and experimentally that any species does not have a single BE on amorphous water surfaces (AWS) but rather a distribution of BEs, which depends on the species and the surface.^16,20−23^ Therefore, the icy grains should be large and varied enough to allow to reconstruct the BE distribution of a species and not just a value. To overcome the aforementioned problems, we have recently proposed^24^ an automatic and unbiased approach to construct water-ice clusters and obtain the binding energy distribution of any species (see Methodology for further details).

In addition to the icy model definition, the second important issue to compute the BE is the adopted level of theory, which always represents a compromise between the computational accuracy and the computational cost (method and system size).

Methods based on molecular mechanics may reach some accuracy when they are designed to treat very specific cases but fail for cases outside their specific parametrization. Alternatively, methods based on the best level of quantum chemistry, such as the gold standard CCSD(T),^25^ ensure a well-balanced treatment of all the relevant interactions responsible for the adsorption on the icy grain surface, irrespective of the considered adsorbate molecule. However, the computational time required by CCSD(T) grows too steeply to be applicable to large icy grains.

Here, we propose a new method that optimizes the computation accuracy on very large icy grain models. Specifically, we implemented an automatic procedure that is based on the ACO-FROST code, recently developed by our group,^24^ to construct a large (⩾1000 water molecules) icy grain. Briefly, only a selected portion of the icy grain, where the adsorption takes place, is treated at a very high level of theory, while the whole cluster is treated at a lower level. This procedure itself is not completely new, as it has been already adopted in the field of surface science adsorption^26^ and for some ice models.^16,27−30^ In a recent work, we adopted a similar scheme to improve the BEs computed for a set of molecules on periodic ice models (both crystalline and amorphous) reaching CCSD(T)-quality results.^16^ Similarly, Duflot et al.^31^ adopted a QM:MM approach using for the QM method the DLPNO-CCSD(T) technique,^32^ a very accurate and computationally feasible version of the CCSD(T) standard based on localized orbitals and the PM6 semiempirical method^33^ for the rest of the system.

Our newly proposed procedure, described in this work, possesses the following novelties with respect to the above works:

(i) an unbiased procedure to generate a large variety of adsorbed structures, not dependent on the nature of the adsorbate molecule and the size of the icy cluster, which allows computation of a BE distribution of the considered species
(ii) the low-level theory adopted to treat the whole icy cluster on the basis of the accurate semiempirical tight-binding xTB-GFN2 method, very recently developed by Grimme’s group^34^
(iii) the high-level theory adopted to describe the ice around the adsorbing site on the basis of the DLPNO-CCSD(T) method with a selection of large Gaussian basis sets

In addition, the procedure is carried out automatically by a package of Python scripts, which allow the construction, submission, and data extraction of the needed calculations.

The BE values resulting from the above approach should in principle be comparable to experimental derivations of BEs. However, this is not straightforward for the following reasons. Binding energies are usually experimentally derived via the so-called temperature-programmed desorption (TPD) method. Strictly speaking, this method provides the desorption activation energy (DAE), which is often interpreted as BE. In practice, the DAE is derived indirectly from the TPD peaks through Redhead method^35^ or more sophisticated numerical techniques. In most TPD experiments, a water-ice surface hosts a monolayer of the adsorbate and, therefore, the BE also depends on the surface coverage.^23^ This renders the comparison between DAE and the computed BE to be actually not straightforward.^36^ For example, ice restructuring processes may affect the final DAE, making it different form the BE. Also, sometimes TPD experiments only provide desorption temperature peaks *T*_des_, with no numerical estimate of the DAE. For instance, Collings et al.^37^ computed the BE of a species X as BE(X) = [*T*_des_(X)/*T*_des_(H_2_O)] × BE(H_2_O), in which *T*_des_(X) is the desorption temperature of the species X in constrast with that of water, *T*_des_(H_2_O), by assuming BE(H_2_O) = 4800 K (∼40 kJ/mol). For the above reasons, a one by one comparison between experiment and modeling should be carried out with extreme care, particularly when a BE distribution is computed, as in the present work.

For our first application of the new method presented here, we chose the ammonia molecule, because it is a thoroughly studied and important species in the molecular ISM. It is the first detected interstellar polyatomic molecule^38^ and one of the most observed, ubiquitous, and studied. It is found in a gaseous form toward the Galactic Center warm molecular clouds and cores,^38,39^ diffuse clouds,^40^ massive hot cores,^41^ molecular outflows,^42^ solar-type protostars,^43^ cold molecular clouds,^44^ prestellar cores,^45^ and protoplanetary disks.^46^ Ammonia is also observed to be quite abundant in the icy mantles that envelope the interstellar dust grains in cold regions.^47^ Obviously, whether ammonia is in either gaseous or solid forms is governed by its BE. Along the same vein, understanding the ammonia chemistry requires a good knowledge of the ammonia BE and, more specifically, its BE distribution, which is the focus of this work.

## Methodology

### Icy Grain Model and NH_3_ Binding Site Sampling

The water-ice grain model used throughout this work, the binding energy sampling procedure, and the preliminary BE optimized structures were taken from a previous work by our group, which is summarized in this section.^24^

#### Water-Ice Grain Model

In order to build up the grain model, a bottom-up approach was followed: i.e., by random successive aggregations of water molecules. A geometry optimization was performed at each addition of a water molecule, followed by a short molecular dynamics (MD) run at 10 K every 10 added H_2_O molecules, to mimic the induced thermal motion due to the partially transferred energy of water formation^19^ occurring in the real grain but not taken into account here (*vide supra*).

As was already discussed in the Introduction, our grain model includes 200 water molecules, large enough to allow for a proper sampling of many adsorbing sites in comparison to previously adopted models. The grain construction was performed with a mixed semiempirical and molecular-mechanics level using the xTB (v.6.3.3)^48^ code (GFN2^34^ and the force field GFN-FF methods^49^) developed by Grimme’s group at the University of Bonn.

#### Binding Energy Sampling Site Procedure

**Figure 1:**
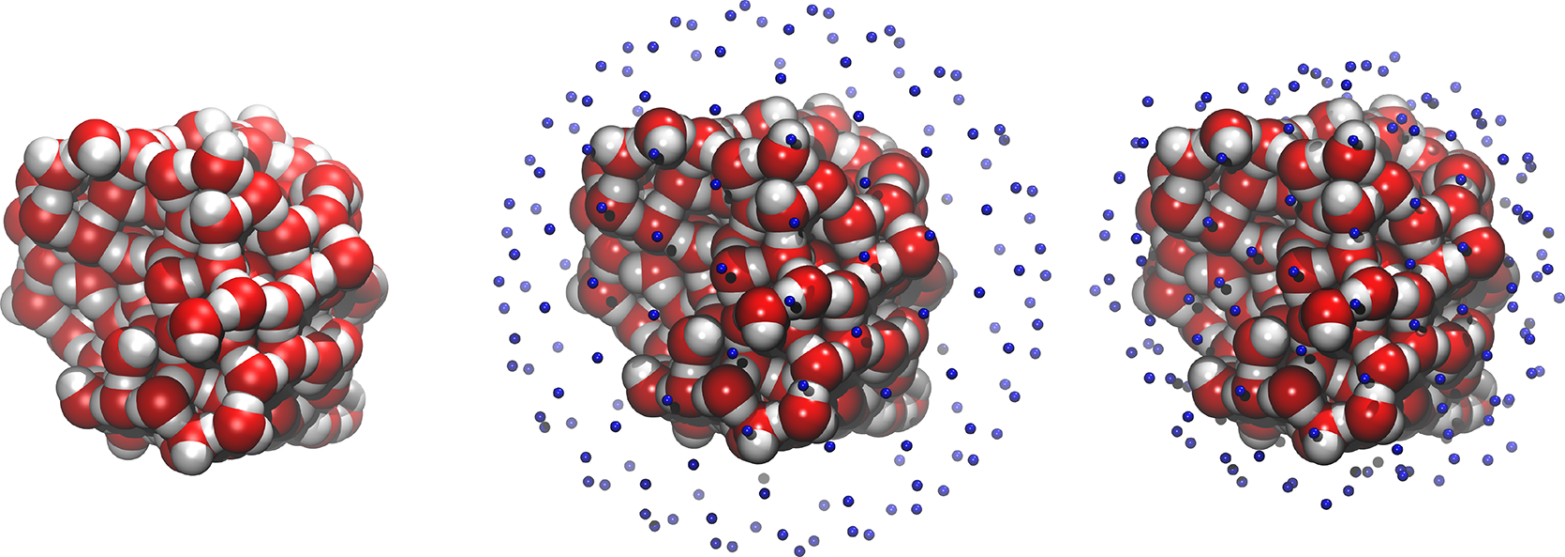
(left) Icy grain model, (center) model with overlapped 162 vertices grid points shown in blue, and (right) model with the same vertices projected closer (2.5–3 Å) to the grain surface. Atom color legend: oxygen in red, hydrogen in white. Data were taken from ref (24).

The NH_3_ binding site sampling was done by placing a grid consisting of 12 vertexes (forming an icosahedron), which were tightened for a total of 162 vertexes uniformly spread around the grain.^50^ The grid points were projected closer to the grain surface, and each point was substituted by a randomly oriented ammonia molecule with respect to the direction vector joining the N atom and the grain center of mass. The projection gives a distance between 2.5 and 3 Å from the grain, used to locate NH_3_ (Figure 1).

#### Preliminary Geometry Optimization

After the NH_3_ sampling, a preliminary geometry optimization via xTB-GFN2^34,48^ was performed. Two subsequent geometry optimizations were carried out in which (i) only the NH_3_ molecule was set free to relax on the grain, while all the water molecules were kept fixed at the optimized free grain positions, and (ii) the atomic positions of NH_3_ and the water molecules included within a cutoff distance of 5 Å from the NH_3_ were relaxed, while the remaining water molecules were kept fixed. This choice enforces the structural rigidity experienced by the water molecules in a real (and much larger) icy grain. During the second task, we found cases where the number of mobile water molecules changed during the optimization procedure, due to the rearrangements of both the NH_3_ and the water molecules within the selected zone. In these cases, the described cycle was repeated by again selecting a new mobile zone and reoptimizing the structure until no changes in the number of water molecules occurred.

### Computational Methods

After a preliminary geometry optimization with the xTB (v.6.3.3)^48^ computational program, the refined binding energy distribution of ammonia on the amorphous ice model was obtained by combing the tools implemented in three codes: xTB (v.6.3.3), Gaussian (v.16, Revision B.01),^51^ and ORCA (v.4.2.1).^52^ We relied on the multilevel ONIOM^53^ (DFT:xTB-GFN2) approach as implemented in the Gaussian program to obtained accurate optimized geometries. As the GFN2^34^ method has not yet been implemented in the Gaussian program, xTB (v.6.3.3)^48^ was used as an external program to work on the low-level zone of the ONIOM method. Finally, the energies of the high-level zone were refined with ORCA (v.4.2.1)^52^ at the DLPNO-CCSD(T)^54^ level of theory. Rendering of molecular images has been obtained via VMD software,^55^ while the graphics elaboration and plots were obtained via the TikZ and PGFPlots LATEX packages.

#### ONIOM Method

The ONIOM (“our own N-layered integrated molecular orbital and molecular mechanics”) method^56^ is a hybrid approach that enables different *ab initio*, semiempirical, or classical mechanics based methods to be combined to different parts of a system to give a reliable hamornic frequencies, geometry and energy at a reduced computational cost. All of the calculations were performed with the two-layer ONIOM(QM:SQM) method. To be specific: the zone in which the quantum-mechanical method (QM) is used (also called the Model zone) consists of NH_3_ and neighboring water molecules within 5 Å from NH_3_, while the whole system (Real zone) is treated at the semiempirical quantum mechanical (SQM) level. The total energy (*E*), gradient vector () and Hessian matrix () for the ONIOM(QM:SQM) two-layer set up are, therefore
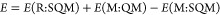
1a
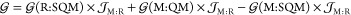
1b
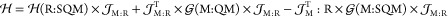
1cwhere  is the Jacobian matrix between the Model (M) and the Real (R) nuclei.

The binding energy (BE, positive for a bounded system), is defined as the opposite of the interaction energy, the last quantity being the difference between the energy of the complex between the grain and the adsorbate (*E*_c_) and the sum of the energies of the isolated adsorbate (*E*_ads_^iso^) and the isolated grain (*E*_grn_^iso^). The equation adopted for the calculation of the ONIOM BEs, after eq 1a, is
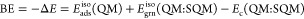
2where the energies of the isolated systems are referred to the specified level at which geometry are also optimized. BE can be decomposed in the pure electronic interaction (BE_e_) corrected for the basis set superposition error (BSSE) and the deformation energy (δ*E*_def_) contributions.

The BE_*e*_ is given by
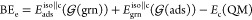
3where  and  are the energies of the isolated adsorbate and the grain in the geometries assumed in the complex (iso∥c) in the presence of the ghost orbitals of the grain  and the adsorbate , respectively. Obviously, as the BSSE is already taken into account by the definition in the GFN2 method, eq 3 only applies to the QM methods (*vide infra*) on the model zone.

The δ*E*_def_ value is given by
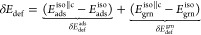
4where δ*E*_def_^ads^ and δ*E*_def_^grn^ are the deformation energy of the adsorbate and the surface, respectively. δ*E*_def_ is for the large majority of the cases a positive quantity; the exceptions will be discussed in a dedicated section.

Moreover, vibrational frequencies were computed on the model zone to obtain the zero-point energies (ZPE), from which the ΔZPE resulted as
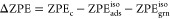
5

When all the aforementioned contributions are included, eq 2 becomes
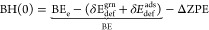
6

In our ONIOM setup, the low-level layer was treated with the xTB-GFN2 semiempirical quantum mechanical (SQM) method,^34^ working as an external program with Gaussian16. The default xTB-GFN2 parameters were used for the SCF. On the high-level layer two different methods were used in order to compute subsequent tasks:

- Geometry optimization and frequency calculations: the B97D3^57,58^ functional, as implemented in Gaussian16, with the aug-cc-pVTZ basis set^59^ and the default setup for geometry optimization, SCF, and integral grid density.
- Final energy refinement: DLPNO–CCSD(T) method,^32,54^ as implemented in ORCA, with aug-cc-pVTZ as the primary basis set, while aug-cc-pVTZ/C^60^ is used as the auxiliary basis set for the resolution of the identity (RI) approximation in electron repulsion integrals. All these calculations were carried out with a tight-PNO set up and the default settings for the SCF.

**Figure 2:**
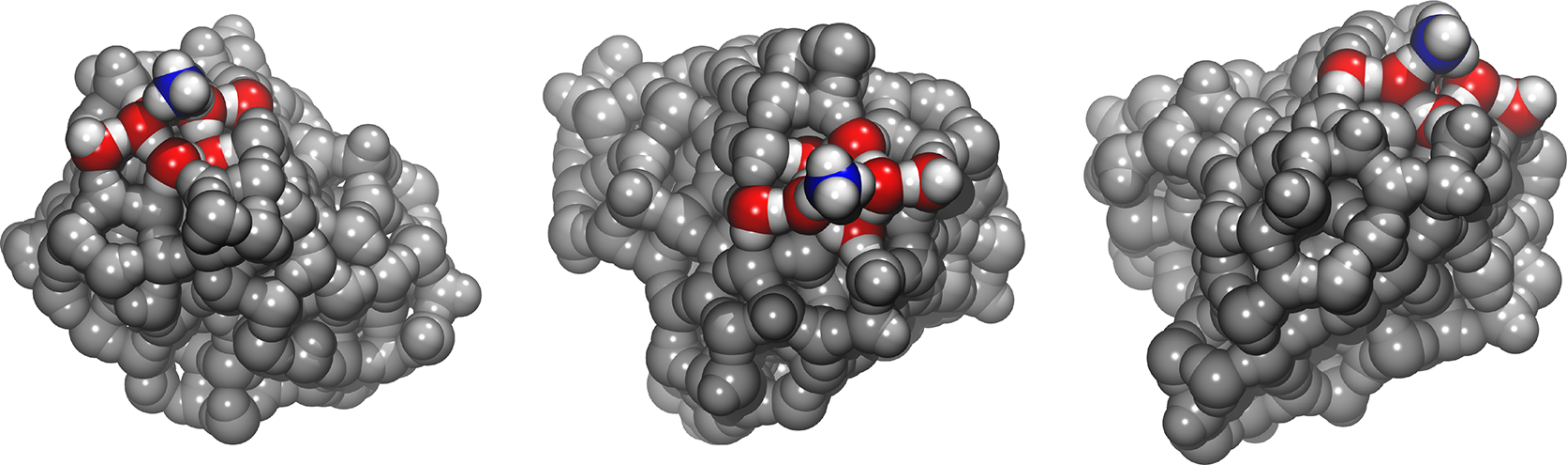
Three different perspectives showing the ONIOM zone: the atoms in the Model (high-level) zone are shown in colors while the low-level zone of the system is pictured in gray. Atom color legend: oxygen in red, nitrogen in blue, hydrogen in white.

During the ONIOM geometry optimization all atoms outside the model zone were kept fixed; only mechanical embedding and no microiterations were used. In the frequency calculations (calculated in the harmonic approximation), only the normal modes related to the nuclei inside the Model zone were taken into account, keeping all the other nuclei fixed (Figure 2).

The treatment of the isolated icy surface required extra care, as the Model zone may change during the search for the optimum structure when the grain is adsorbing the NH_3_ molecule. Therefore, to ensure a proper coherence we used in this section as Model zone for evaluating the energy *E*_grn_^iso^ of the free grain, the very last set of water molecules defined in the cyclic procedure described above, on an otherwise unique and fixed reference geometry of the free cluster.

### Model Zone Setup

The definition of the Model zone, which is the core of any ONIOM-based procedure, implies not only a proper choice of the level of theory but also the number of atoms to be included in the QM description.

#### Geometry Optimization Constraints

We adopted the same strategy for the ONIOM calculation used for the optimizations performed with the GFN2^34^ level. However, since the method to treat the Model zone is computationally demanding, a less tight criterion on the optimization convergence was used: when the number of water molecules of the Model zone changes by ⩾|2| units, we run further geometry optimizations with the redefined Model zone, until the above condition is satisfied.

#### Model Zone Size Benchmark

The Model zone defined within 5 Å from the NH_3_ relies on a tradeoff between two main requirements: (i) including all of the local NH_3_–H_2_O interactions and (ii) saving computational resources.

In order to understand the influence of the Model zone size on the BE, a benchmark was performed by taking the single-point energy evaluation of eight different optimized cases with the standard Model zone definition (5 Å) and expanding its size from 5 Å up to 8.5 Å (which corresponds to including up to 21–34 water molecules) while the geometry of the whole system was kept fixed.

**Figure 3:**
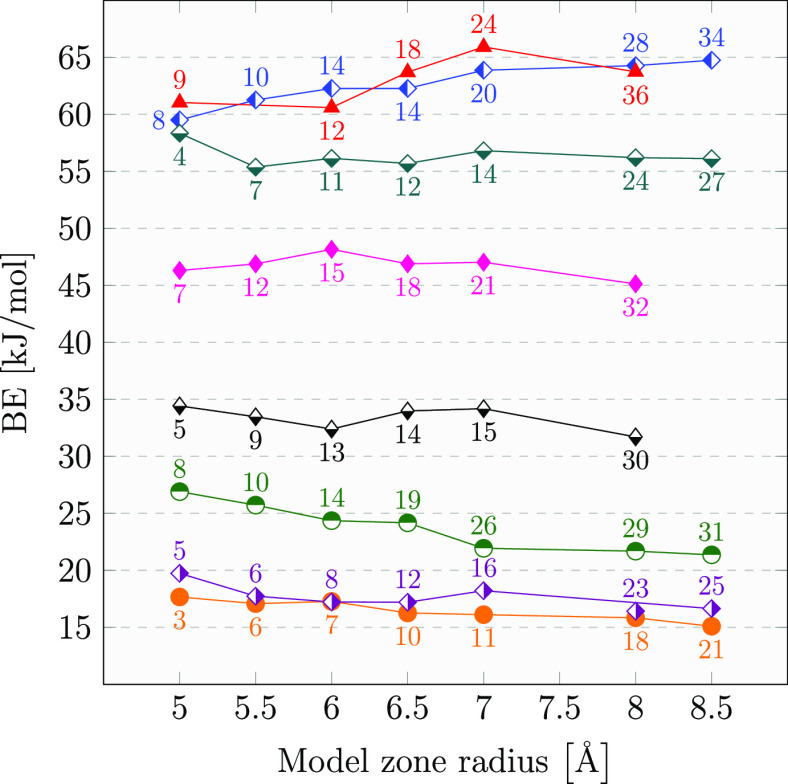
BSSE-corrected BEs calculated at the ONIOM(B97D3/aug-cc-pTVZ:xTB-GFN2) level as a function of the Model zone size. Each symbol/color represents the same BE sample, while the number of water molecules inside the Model zone is reported close to the related symbol with the same color.

Single-point energy calculations were carried out at the same level of theory described in the previous section: i.e., ONIOM(B97D3/aug-cc-pVTZ:xTB-GFN2). Figure 3 shows, for all but two samples, a change in the BE value well within 5 kJ/mol and a rather flat variation in the BE values. The two exceptions are at the limit of the threshold of 5 kJ/mol (i.e., within the chemical accuracy limit).

#### Model Zone Method Benchmark

**Figure 4:**
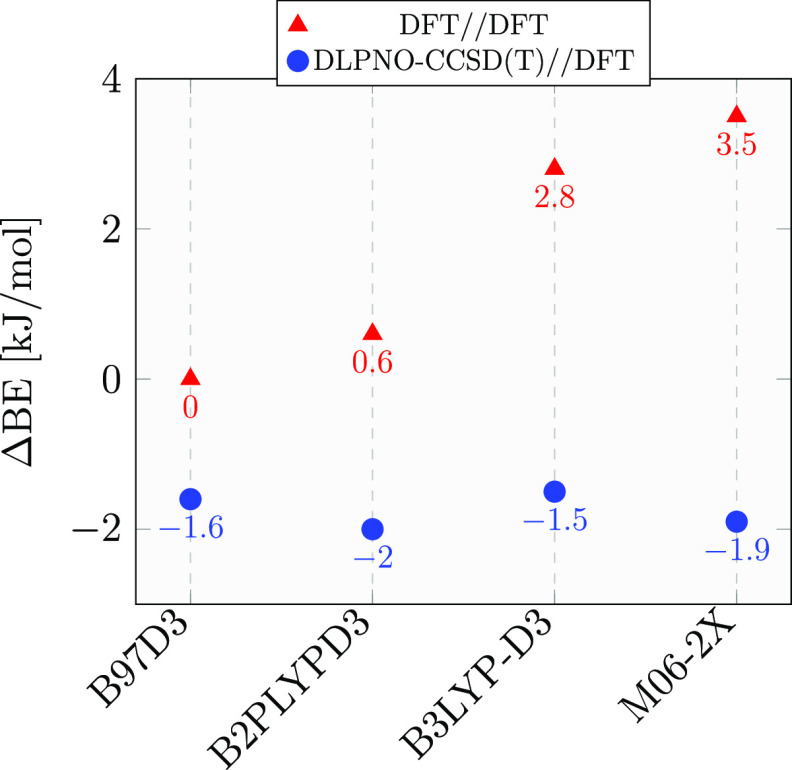
Differences ΔBE between the BE B97D3 reference value (48.4 kJ/mol) and the BEs computed with the reported QM methods, all coupled with a aug-cc-pVTZ basis set quality and BSSE correction.

The pure GGA B97D3 functional^57,58^ used to deal with the Model zone is well adapted to deal with noncovalent interactions such as those responsible for the grain cohesion and the NH_3_ BEs.^57^ To assess the B97D3 performance for the present case, we compared, for one selected NH_3_/grain case, structures and BEs (corrected for BSSE) with (i) the B2PLYPD3 double-hybrid functional with empirical dispersion corrections,^61^ (ii) the B3LYP^62,63^ functional with the D3 version of Grimme’s dispersion with the Becke–Johnson damping function,^58^ and (iii) the Minnesota double-exchange M06-2X functional,^64^ coupled with the aug-cc-pVTZ^59^ basis set. DFT BEs were then refined at the DLPNO-CCSD(T)/(aug-cc-pVTZ and aug-cc-pVTZ/C) tight-PNO level (all the values were corrected for the BSSE) computed at each DFT optimized geometry. The results are presented in Figure 4. Among all adopted functionals, B97D3 is the one with the closest BE value with respect to the reference DLPNO-CCSD(T) value.

We also calculated the BE with the gold standard CCSD(T)/aug-cc-pVTZ on the same ONIOM(B97D3:xTB-GFN2) sample used in the previous test. The BE relative errors of B97D3 and DLPNO-CCSD(T) with respect to CCSD(T) are 0.9 and −0.7 kJ/mol, respectively. These results validate the performance of both B97D3^57^ and DLPNO-CCSD(T).^65,66^

### Adsorption Site Redundancy Reduction

During the geometry optimization different NH_3_ starting points may end up in the same minimum of the potential energy surface (PES), due to the complexity of the PES and the relatively weak interaction energy. For instance, many identical structures differ only in the permutation between the ammonia hydrogen atoms. Therefore, this redundancy in the adsorption sites was reduced by comparing the RMSD and ΔBE values between all considered structures and discarding the cases for which RMSD ⩽ 1 Å and |ΔBE| ⩽ 1 kJ/mol. After cleaning, a total of 77 unique structures from the total 162 starting points were analyzed.

### Machine Learning Binding Energy Classification

Once the BE distribution, without site redundancy, was obtained, a clustering procedure was performed to analyze the data. Cluster analysis, or clustering, is an unsupervised machine-learning technique that involves the grouping of data points. This grouping is done in such a way that the members of the same cluster can be considered “*similar*” in some way (e.g., through metrics such as the L^2^ distance). In our case we exploited hierarchical agglomerative clustering (HAC), where a hierarchy of clusters is built with a bottom-up approach: each observation starts in its own cluster, and pairs of clusters are merged as one moves up the hierarchy. Sets of observations are linked via the so-called linkage criterion. The algorithm will merge the pairs of cluster that minimize this criterion. In our study we used the Scikit-Learn implementation of HAC,^67^ using the minimum-distance linking criterion (namely “*single*”), specifying an *a priori* number of clusters of 2 (i.e., the number of clusters that we want to find). Finally, we scaled every feature to [0,1] in order to obtain the scaled invariance.

## Results and Discussion

NH_3_ usually behaves as a strong hydrogen bond acceptor, due to the negative electrostatic potential in the nitrogen lone pair region, while being a very weak hydrogen bond donor. For instance, the NH_3_ crystal structure^68^ shows only very weak hydrogen bonds between the NH_3_ molecules, the N···H distance being as great as 2.35 Å. Indeed, our results basically show NH_3_ acting as a strong H-bond acceptor of the dangling hydrogens of the icy grain and a weak H-bonding donor toward the water oxygen dangling atoms. After the harmonic frequency analysis, 16 samples show only one imaginary frequency in the [−50,–8] cm^–1^ wavenumber range. Since the imaginary frequencies fall at very low wavenumbers and do not reflect nuclear motion of the NH_3_ position, we also kept these structures to improve the statistics of the BE distribution, as their very low values do not alter the final BH(0) values.

### NH_3_ Desorption Rate Prefactor

In the desorption process, the desorption rate can be expressed as *k*_des_ = ν(*T*)e^–(BE)/(*k*_B_*T*)^, where ν(*T*) is a pre-exponential factor that takes into account entropic effects, while the enthalpic contribution is inside the exponential part. In order to give reliable data to be used in astrochemical models and/or to have a connection with experiments, a pre-exponential factor must be provided together with the BE. Usually, depending on the substrate and adsorbate, a value between 10^12^ and 10^13^ s^–1^ is assumed in experiments or as a first approximation in modeling studies, as reported by Hasegawa and Herbst^69^ (see e.g. the discussion in Minissale et al.^70^). We prefer to adopt the transition state theory within the immobile adsorbate approximation^70,71^ to estimate the prefactor
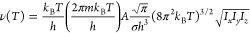
7where *k*_B_ is the Boltzmann constant, *m* is the mass of the molecule, *h* is the Planck constant, *A* is the surface area per adsorbed molecule usually assumed to be 10^13^ N_a_/Å^2^, *I*_*i*_ is the *i*-esimal adsorbate principal moment of inertia, and σ is the symmetry adsorbate rotation factor. For NH_3_, the principal moments of inertia are 2.76, 1.71, and 1.71 amu × Å^2^, σ = 3, and *m* = 17 amu. When these values and a desorption peak at *T*_des_ = 100 K are used, the pre-exponential factor is 1.94 × 10^15^ s^–1^.^70^ This value is recommended in association with the BE values computed with quantum mechanical approaches similar to those described in the present work.

### BE Evaluation: Calorimetric versus TPD Reference

In the BE calculations, the definition of the “free” grain structure, from which the *E*_grn_^iso^ value is computed, is crucial and may differ depending on what process one is simulating, while that for the NH_3_/grain adduct (*E*_ads_^iso^) is unambiguous. Usually, in dealing with adsorption on extended surfaces of metal or oxide materials, the reference structure is the bare isolated surface, fully optimized at the given level. In such cases, the forces keeping the metal atoms or the ions in place are much stronger than the BE with the adsorbate and, therefore, the whole structure is little affected by the interaction. In the present case, the icy grain is held by forces of the very same nature as those occurring between the adsorbate and the water molecules within the grain. Therefore, it may happen that, during the geometry optimization of the adsorbate/grain complex, the grain structure will be altered in such a way that the deformation energy δ*E*_def_^grn^ = *E*_grn_^c^ – *E*_grn_^iso^ becomes negative: i.e., the *deformed grain* is more stable than the isolated starting grain. In other words, the geometry relaxation induced by the adsorbate brings the icy cluster in a new local minimum, slightly deeper than the initial minimum. This only happens in a few cases, especially when the Model zone is redefined due to large movements associated with the NH_3_ molecule. To solve this ambiguity in the definition of the deformation energy, we chose, as a starting structure for the isolated cluster to be optimized, the structure resulting after the interaction of NH_3_. In this way, δ*E*_def_^grn^ will always be positive. We defined these two approaches by considering different reference pristine grain geometries, as “calorimetric” (original initial grain geometry) and “TPD” (reference grain geometry after adsorption), respectively. The BE distributions from the two approaches will be presented and discussed in the following.

**Figure 5:**
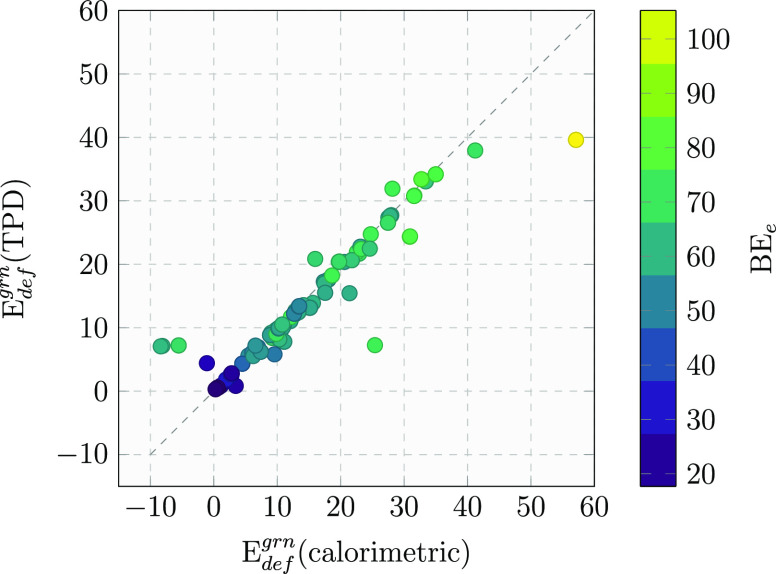
Correlation of TPD vs calorimetric deformation *E*_def_^grn^ energies. The color map shows the corresponding electronic interaction BE_e_ (see eq 6) associated with each point. All data are given in kJ/mol.

In the calorimetric approach, as in microcalorimetric measurements, it is assumed that the reference system is a clean unperturbed surface and that the heat of adsorption occurs when the adsorbate arrives on the surface from the gas phase. In the temperature-programmed desorption (TPD), the molecule is first adsorbed on the surface and then the temperature is raised up to the point at which the adsorbate leaves the surface. Clearly, when the surface is made by water ice, what is left after desorption cannot be considered equivalent to an unperturbed pristine icy surface, as in the calorimetric approach. These two approaches may lead to different BE values, as shown in Figure 5, which correlates the deformation energy *E*_def_^grn^ contribution to the BE computed with both the TPD and calorimetric approaches. The purely electronic BE_e_ (which is free from the deformation energy) is also shown as a reference color bar. As expected, the two approaches lead to the same results for most cases. Nevertheless, there are some exceptions, such as some samples with low deformation energy values, in which the surface restructuring leads to a negative deformation energy in the calorimetric approach. The other two outliers (*E*_def_^grn^(calorimetric) ≈ 25 and 60 kJ/mol) are due to the formation/breaking of some H bonds at the interface between high- and low-level zones, thus implying a redefinition of the Model zone itself and, therefore, the displacements of many water molecules. In the following, we only refer to the TPD method to compute the final BE distribution.

### Binding Energy Distribution

#### New BE Distribution versus Previous Values

**Figure 6:**
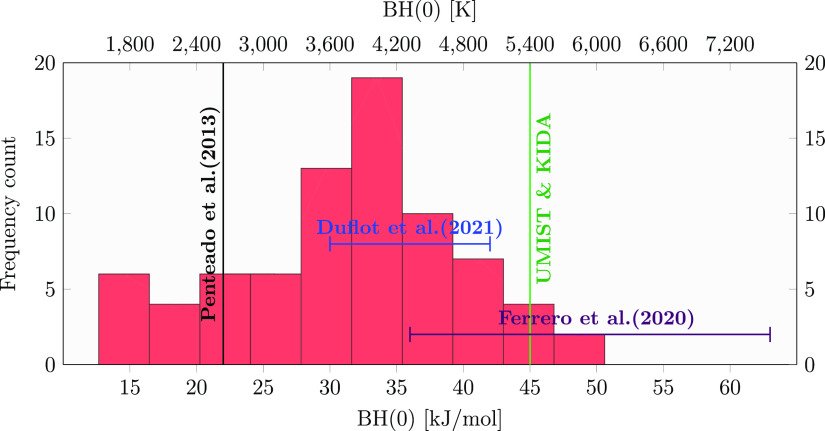
BSSE-corrected BH(0) distribution at the DLPNO-CCSD(T)/aug-cc-pVTZ level and ZPE calculated at the ONIOM(B97D3/aug-cc-pVTZ:xTB-GFN2) level.

The final BH(0) values (see eq 6) have been organized in a bin width distribution following the Freedman–Diaconis estimator,^72^ as shown in Figure 6. Due to the large number of different adsorbing sites the distribution is asymmetric, with a data dispersion ranging from 12.7 to 50.6 kJ/mol and a mean and mode (the most frequent values) of 31.1 and 33.5 kJ/mol, respectively. A fine analysis of the data shows that the deformation energy is the main source of data dispersion. The ZPE plays a minor role in the BH(0), its contribution being on the order of 10% on the total BH(0). The ZPE correction decreases the BE value by about 10 kJ/mol. A value of ∼45.7 kJ/mol is reported in the astrochemistry databases, which is in the same range, or higher, with respect to the BE values for water self-adsorption.^14,73^

A comparison with computed literature BE values by Ferrero et al.,^16^ computed on a periodic crystalline proton-ordered ice slab model (51.8 kJ/mol) and on an amorphous water slab model (35.9–62.8 kJ/mol), is shown in Figure 6. In that work, the sampling of binding sites on the amorphous slab included just seven cases and all the interactions found displayed NH_3_ as an acceptor of at least one hydrogen bond. In the work by Duflot et al.,^31^ a procedure similar to the present one (ONIOM(CBS/DLPNO-CCSD(T):PM6)//ONIOM(ωB97X-D/6-31+G**:PM6)) was adopted to compute a ZPE-corrected BE. BE values of 35.9 ± 11.6 kJ/mol have been computed, in good agreement with our values of 31.1 ± 8.6 kJ/mol, despite the fact that a very different methodology was adopted to build up the underneath ice.

#### Clustering Analysis

On the final data set of 77 BEs, a machine-learning (ML)-based procedure was used in order to correlate BH(0) with other energetic and geometrical parameters:

- the minimum H-bond distance,
- the H-bond angle  referenced to the  H bond
- the deformation energy δ*E*_def_
- the pure electronic BE_e_

**Figure 7:**
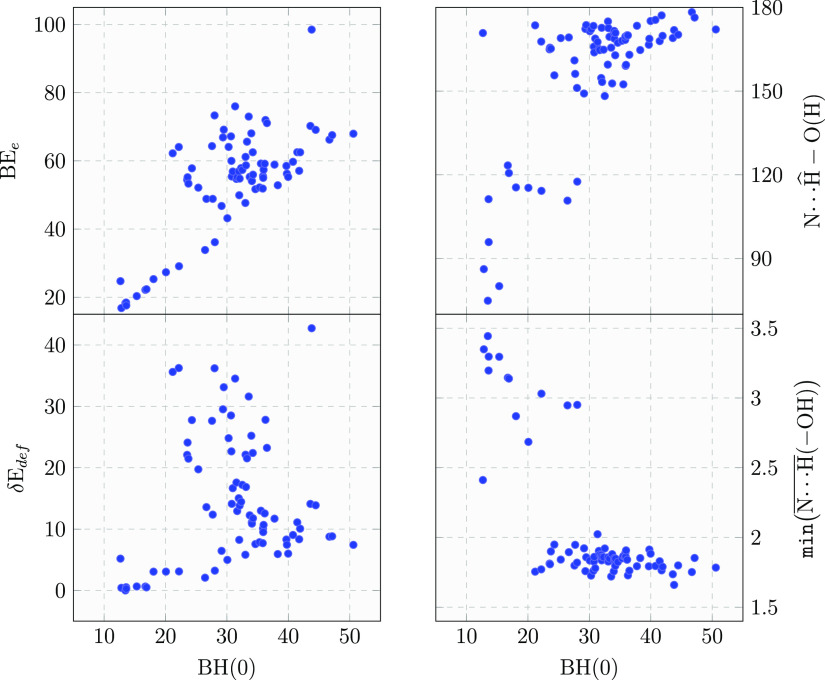
Correlation plots between BH(0) and the feature vectors used in the ML clustering. BH(0), BE_e_ and δ*E*_def_ are given in units of kJ/mol. Distances are given in Å and angles in degrees. All BH(0) and BE_e_ values are BSSE-corrected.

The correlation plots are shown in Figure 7.

The plot of both  and  revealed a rather clean clustering, in which at high BH(0) values correspond to H-bond lengths well below 2 Å (NH_3_ as H-bond acceptor), while at low BH(0) values H-bond distances were over 2.5 Å (NH_3_ as H-bond donor). This correlates also with the  angle, moving from values close to linearity for high BH(0) values to random values from linearity for the low-BH(0) range. Less trivial is the correlation between BH(0) and its different energy components. About the deformation energy δ*E*_def_, a number of points are almost aligned as a baseline in the 0–10 kJ/mol range, while in the region of intermediate BH(0) values the points are quite spread out. The same erratic trend is observed in the correlation with BE_e_, revealing that the vast majority of cases exhibits a final BH(0) value that is a compromise of a large geometry deformation energy compensated by a large electronic binding energy. The few cases at very high BH(0) characterized by small δ*E*_def_ values are due to favorable adsorption sites, already suitable to host the NH_3_ molecule and, therefore, not requiring a large structural deformation.

**Figure 8:**
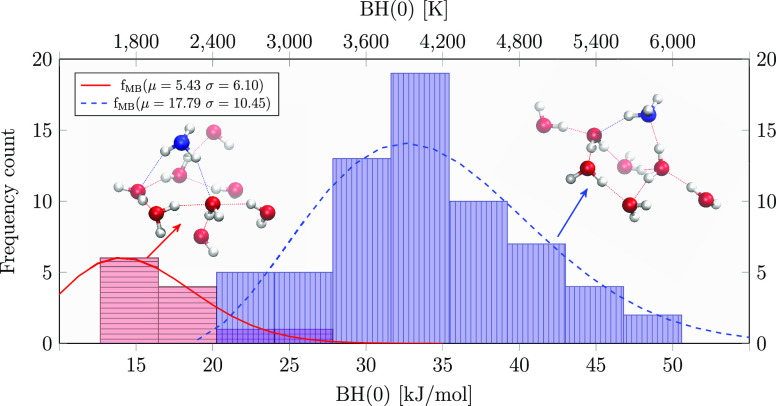
ML clustering analysis applied to the BH(0) distribution of Figure 6. The continuous red and dashed blue curves are the *f*_MB_(hist(BH(0)),σ,μ) Maxell–Boltzmann best fit for the two histogram clusters. The insets show the Model (high-level) zones of two representative samples, with high (rightmost) and low (leftmost) BH(0) values. Atom color legend: oxygen in red, nitrogen in blue and hydrogen in white.

The geometrical clustering analysis applied to the binding energy distribution shown in Figure 6 is reported in Figure 8. The two clusters rely, as expected from chemical knowledge, on the two possible H bonds that the ammonia can form with water: the stronger N···H(−OH) and the weaker N–H···O(H_2_), where the ammonia is respectively a H-bond acceptor and a H-bond donor. In light of these results, the asymmetric shape of the distribution at low BH(0) is due to the cluster distribution related to the H-bonds in which ammonia is the proton donor.

Moreover, as shown in Figure 8, the two histogram clusters were fitted with a non-normalized Maxwell–Boltzmann distribution function *f*_MB_(*x*,σ,μ):
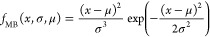
8where, in our case, *x* values are the bin width medium of the BH(0) histogram and μ and σ the distribution parameters.

**Figure 9:**
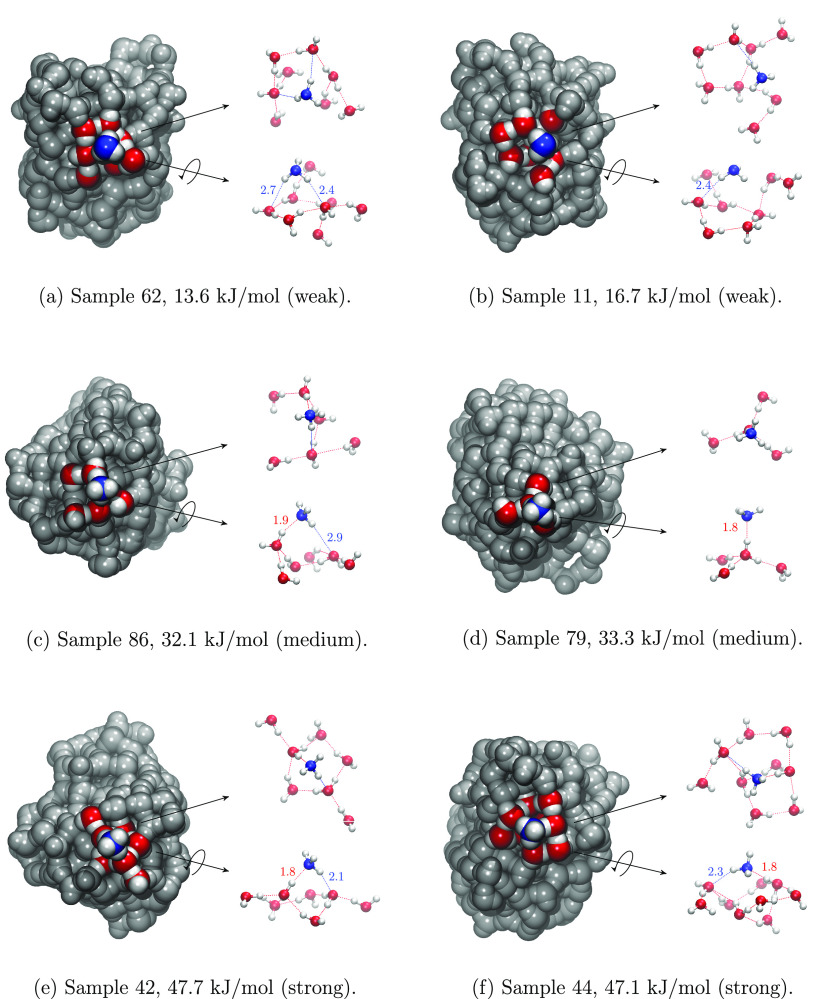
Selected cases of weak, medium, and strong NH_3_ BH(0) values. On the right of each cluster the Model zone is highlighted in ball and stick representation (top and side views). Distances are given in Å. An online database could be used to easily interact with and inspect all the samples, as described in the relative subsection.

Figure 9 shows a selected number of grain/NH_3_ structures, spanning from weak to strong values of BH(0), evidencing the already mentioned features of NH_3_ when it interacts through H bonds.

Experimental evidence of the tail distribution at very low BE(0) can be searched in the literature, as summarized by Ferrero et al.:^16^ NH_3_ TPD experiments on amorphous and crystalline water surfaces were reported by Collings et al.^37^ and He et al.^23^ However, Collings et al.,^37^ who only carried out experiments on amorphous water ice, did not explicitly derive the NH_3_ BE. On the basis of their curve, Penteado et al.^13^ successively estimated a BE equal to 22.5 kJ/mol = 2706 K using a pre-exponential factor equal to 10^12^ s^–1^. The BE becomes 3460 K if a pre-exponential factor of 1.94 × 10^15^ s^–1^ is used. In contrast, He et al.^23^ only derived the BE for adsorption on crystalline ice, as they found that NH_3_ desorbs at the temperature where the amorphous water ice becomes crystalline. Inverting the TPD curve for the crystalline ice adsorption using the pre-exponential factor of 10^–12^ s^–1^, He et al.^23^ derived a BE of about 4000 K for a low surface coverage (⩽0.5) and of about 3000 K for a full a coverage (see Figure 9 of ref (23)). However, this last value is almost the same as that derived by the TPD experiments of NH_3_ adsorbed on a gold surface,^74,75^ suggesting that a sizable fraction of BE is due to the lateral interactions between NH_3_ within the adsorbed multilayers and not to the interaction with the ice surface. Moreover the 3000 K BE value (computed with a pre-exponential factor of 10^–12^ s^–1^) becomes 3754 K, with a pre-exponential factor of 1.94 × 10^15^ s^–1^, indeed larger than our lower-end BE value. Therefore, in both the experimental works presented, the low end of the ammonia BE that we computed was not detected. One possibility is that, under low NH_3_ coverage, NH_3_ exhibiting very weak BE values (such as that corresponding to our lowest BEs) will easily diffuse to empty sites characterized by higher BE values, instead of being entirely desorbed. This process is only effective at moderate NH_3_ coverage, where sites with high BE values are still available for occupation. This indeed happens in the TPD experiment, in which the thermal heating brings an oversampling of sites at high BE values.^70^ While a detailed astrochemical modeling that may better elucidate this point is postponed to a dedicated work, this discussion also highlights how critical the comparison can be between experimental data extracted from the TPD and the computed data through quantum mechanical calculations if the pre-exponential factor is not treated on the same foot and similar NH_3_ surface coverages are considered.

### xTB-GFN2 Validation

**Figure 10:**
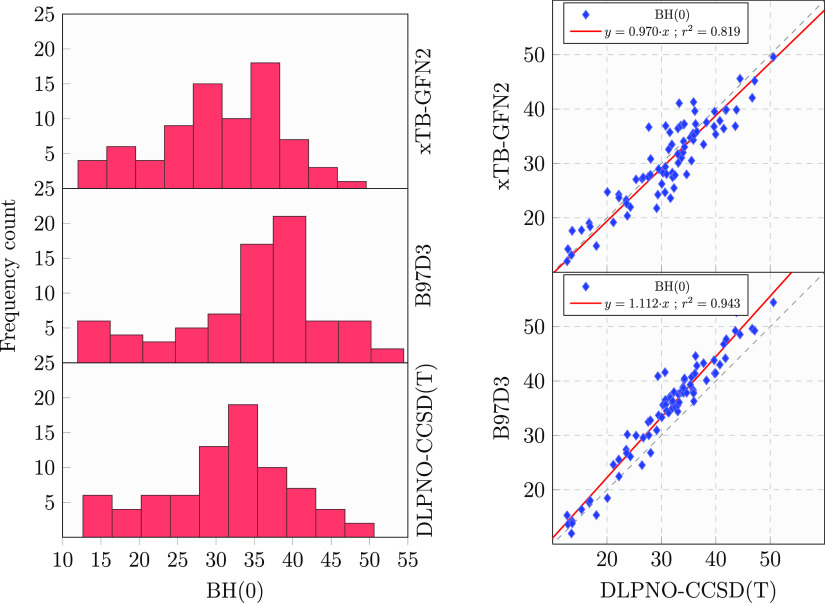
(left) BH(0) distributions for DLPNO-CCSD(T), B97D3, and xTB-GFN2 methods. (right) BH(0) correlation diagrams of B97D3 and xTB-GFN2 against DLPNO-CCSD(T). Each histogram bin width has been calculated with the proper Freedman–Diaconis estimator. All values are given in in kJ/mol.

In our recent works,^24,76,77^ we adopted xTB-GFN2 as the low-level semiempirical method. The ONIOM procedure requires, to be robust, a low level of theory giving structures and energies not too far from the high-level theory. Here, we compare the xTB-GFN2 BH(0) values computed as a single-point xTB-GFN2 energy evaluation on the ONIOM optimized geometries with the more accurate ONIOM structures, computed at the DLPNO-CCSD(T) level. Figure 10 shows the excellent performances of xTB-GFN2, considering its very low computational cost, also in comparison with B97D3, which gives results in better agreement with the DLPNO-CCSD(T) data. xTB-GFN2 BH(0) values are, instead, systematically underestimated with respect to the reference. The worse GFN2 correlation may be due to the geometric distortion in the Model zone, since it is evaluated at the B97D3 level.

### Astrochemical Implications on NH_3_ BE Distribution

**Figure 11:**
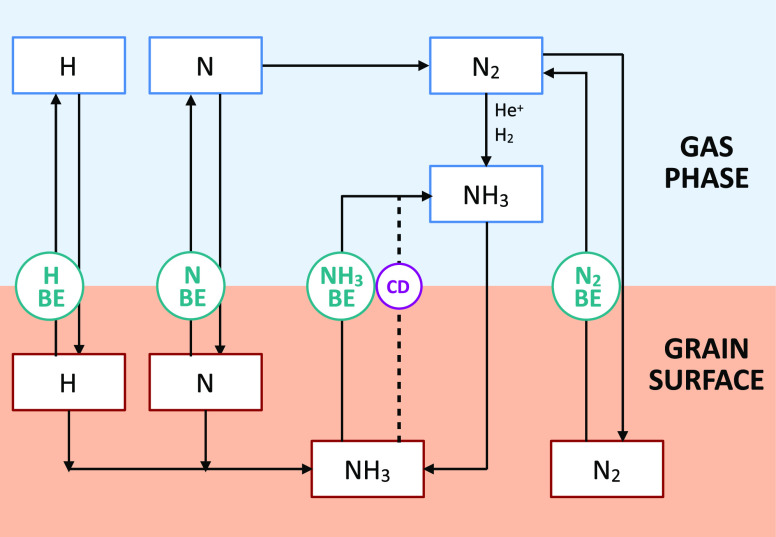
Scheme of the interstellar chemistry involving NH_3_. Ammonia can be synthesized on the grain surfaces by hydrogenation of frozen N^79^ (left part of the figure) or in the gas phase from reactions involving N_2_^78^ and then frozen onto the grain surfaces (right part of the figure). Once on the grain surface, NH_3_ can be thermally desorbed or injected into the gas phase via the so-called chemical desorption (CD) or because of the cosmic-ray desorption (CRD), as marked with the dashed line. Both thermal and CRD desorption are governed by the NH_3_ BE and involve the whole frozen NH_3_, while CD injects a small fraction (⩽1%) of the NH_3_ formed by the N hydrogenation on the grain surface.

As mentioned in the Introduction, NH_3_ is ubiquitous in the molecular ISM and can be either gaseous or iced. Also, NH_3_ can be formed both in the gas phase from molecular nitrogen^78^ and on the grain surfaces by hydrogenation of atomic nitrogen,^79^ as shown in Figure 11.

The crucial parameter that governs whether NH_3_ is in the gaseous or solid form is its BE. The fact that the NH_3_ BE is not a single value but a distribution that covers a relatively large range of energies, from 1800 to 6000 K (15–50 kJ/mol), can have an important effect (see e.g. Grassi et al.^80^).

While gaseous ammonia in warm (⩾100 K) regions does not present any particular puzzle, its presence in cold objects might. The most extreme example is the gaseous ammonia observed in prestellar objects. In L1544, a very well studied prestellar core,^81^ the dust temperature at the center of the condensation is only 7 K^82^ and ammonia should be completely frozen onto the grain mantles.^83,84^ In contrast, ammonia is observed to be gaseous.^45^ Various reasons have been proposed, mainly that ammonia is desorbed from the grain mantles because of the chemical energy released by its formation, which is believed to be due to the hydrogenation of N (see e.g. Sipilä et al.^84^). These authors found that slightly less than 1% of the ammonia formed on the grain icy surfaces could be necessary to reproduce the observed values. However, these authors also modeled the possibility that the ammonia BE is smaller than the standard high value and considered the cases with BEs equal to 1000 and 3000 K (8 and 25 kJ/mol), respectively. As expected, an ammonia BE equal to 1000 K would result in an overly large gaseous ammonia abundance with respect to the observed value. However, if one considers the BE distribution of Figure 8, about 3% of the frozen ammonia would have a BE equal to 1800 K (15 kJ/mol) so that, very likely, the predictions would be in agreement with the observations.

## Summary and Conclusions

In this paper we provide a new framework to compute the binding energy (BE) distribution of any relevant interstellar species adsorbed at the surface of an icy grain mantle, in a reproducible and user-friendly automated way. Two main parameters are controlled by the user: the ONIOM high-level zone size, which should be large enough to account for all the H-bond interactions with the ice, and the DFT method for geometry optimization (and subsequent frequency analysis). The framework can be divided into four subsequent blocks:

(1) building up of the grain model and choice of the species to be absorbed
(2) sampling of all possible binding sites on the icy grain model by an automatic unbiased procedure and geometry optimization with a low level of theory (xTB-GFN2)
(3) geometry optimization and zero-point energy correction using the ONIOM method (B97D3:xTB-GFN2)
(4) final ONIOM single-point (SP) energy refinement with a higher level of theory (DLPNO-CCSD(T)//B97D3:xTB-GFN2)

The first two tasks are encoded in the ACO-FROST program^24^ (see also Icy Grain Model and NH3 Binding Site Sampling subsection). An extensive benchmark applied to the ammonia case is reported in Methodology, where we demonstrate the performance of the chosen methodology, highlighting its excellent compromise between accuracy and computational cost. Moreover, we also demonstrated in a dedicated section that the same distribution calculated at the full xTB-GFN2 level is similar to that at the ONIOM(DLPNO-CCSD(T)//B97D3:xTB-GFN2) level, which confirms the robustness of GFN2 despite the fact that its cost is orders of magnitude smaller than those of DFT and DLPNO-CCSD(T).

We highlight a particular aspect that needs to be treated with particular care: the reference of the bare water grain. This attention is due to the cooperativity and mobility of the H-bond network that, when the bare grain is optimized after removing the adsorbate, can lead to strong rearrangements which may result in a negative deformation energy (which is almost always a positive quantity). For this reason, we propose and compare two different references for the bare icy surface, which somehow mimic the two experimental techniques used to study such a phenomenon: TPD (each reference is obtained after adsorption, i.e. the NH_3_ and the bare grain structure reoptimized) and calorimetry (the reference is the starting optimized bare grain, before site sampling).

The final ZPE- and BSSE-corrected BE distribution (BH(0)) for ammonia shows, as expected from our 77 unbiased samples, all the possible interactions of NH_3_ with a water surface, acting as a H-bond donor and/or acceptor. This variety of BE is made possible by the large number of chemically different binding sites that the built icy grain model presents (not only in terms of dangling species but also from a morphological point of view of the global structure). Using an unsupervised machine-learning clustering technique, we correlate the structures and their BH(0). The two clusters found with the ML algorithm can be approximated by two Maxwell–Boltzmann distribution functions with the first peak at around 34 kJ/mol (or ∼4000 K) and the second peak at ∼15 kJ/mol (or ∼1800 K). As expected, the asymmetrical shape at low BH(0) is due to ammonia acting as a H-bond donor, while at high BH(0) we found ammonia acting as both donor and acceptor from a variety of ice dangling hydrogen atoms whose propensity to make H-bonds is modulated by the cooperativity of the H-bond network within the grain. The first peak of the NH_3_ BH(0) distribution matches very well with the data in the literature, from both experimental and theoretical works. In contrast, we show for the first time the presence of a second peak at lower BH(0). We discuss how this second peak may explain the longstanding puzzle of the presence of ammonia in cold and dense ISM.

In summary, the major novelty of our work is the development of a framework with a general applicability to simulate all statistically meaningful binding sites of a species adsorbed on an icy surface, with high accuracy at reasonable computational cost.

It allows producing realistic BE distributions of interstellar molecules, which is a breakthrough with important implications in astrochemistry. Our results point toward a more complex scenario about BEs than it has been thought in the past, as BE in astrochemical models are very often assumed to have a single or very few values, which is an oversimplification of the reality.

Finally, the presence of a low BE definitively has an important effect on our understanding of the chemical evolution of the molecular ISM.

### Online Database

To easily handle the large data set of BE samples (atomic coordinates and BH(0) values), we developed and made publicly available a Web site^85^ based on the molecule hyperactive JSmol plugin (Jmol: an open-source Java viewer for chemical structures in 3D). The extended electronic version of the calculated results, the 77 optimized structures at the ONIOM(B97D3/aug-cc-pVTZ:xTB-GFN2) level, are available at https://tinaccil.github.io/Jmol_BE_NH3_visualization/.

## Supporting Information Available

Dataframe.csv presenting all the binding energy (BE) information for each sample, where all the values are referenced to the TPD approach (see the discussion in the paper), all the of computed structures (.xyz files) at the ONIOM(B97D3/aug-cc-pVTZ:xTB-GFN2) level, in the structures folder, with the complex structures (grain_molX.xyz) and the Model zone (CP_grain_molX.xyz), the number inside (*X*), and the file name referenced to the sample (“n_sample”, in the Dataframe), bare grain and ammonia (bare_grain.xyz and nh3.xyz), optimized respectively at xTB-GFN2 and B97D3/aug-cc-pVTZ, input examples for all of the programs in the example_input folder, and the script to interface (i.e., xtb-Gaussian.sh) xtb to Gaussian16 (ZIP)
